# Experiences of Living with Severe Chronic Fatigue Syndrome/Myalgic Encephalomyelitis

**DOI:** 10.3390/healthcare9020168

**Published:** 2021-02-05

**Authors:** Victoria Strassheim, Julia L. Newton, Tracy Collins

**Affiliations:** 1 CRESTA Fatigue Clinic, Newcastle upon Tyne Hospitals NHS Foundation Trust, Newcastle upon Tyne NE4 6BE, UK; Julia.Newton@newcastle.ac.uk; 2 Academic Health Science Network–North East and North Cumbria, Newcastle upon Tyne NE4 5PL, UK; 3 Population Health Science Institute, Newcastle University, Newcastle upon Tyne NE1 7RU, UK; 4 Department of Social Work, Education and Community Wellbeing, Coach Lane Campus, Northumbria University, Newcastle upon Tyne NE7 7AX, UK; tracy.l.collins@northumbria.ac.uk

**Keywords:** ME/CFS, severe, very severe, housebound, qualitative, interview, experience

## Abstract

Chronic Fatigue Syndrome/Myalgic Encephalomyelitis (CFS/ME) is a rare disease with no known etiology. It affects 0.4% of the population, 25% of which experience the severe and very severe categories; these are defined as being wheelchair-, house-, and bed-bound. Currently, the absence of biomarkers necessitates a diagnosis by exclusion, which can create stigma around the illness. Very little research has been conducted with the partly defined severe and very severe categories of CFS/ME. This is in part because the significant health burdens experienced by these people create difficulties engaging in research and healthcare provision as it is currently delivered. This qualitative study explores the experiences of five individuals living with CFS/ME in its most severe form through semi-structured interviews. A six-phase themed analysis was performed using interview transcripts, which included identifying, analysing, and reporting patterns amongst the interviews. Inductive analysis was performed, coding the data without trying to fit it into a pre-existing framework or pre-conception, allowing the personal experiences of the five individuals to be expressed freely. Overarching themes of ‘Lived Experience’, ‘Challenges to daily life’, and ‘Management of the condition’ were identified. These themes highlight factors that place people at greater risk of experiencing the more severe presentation of CFS/ME. It is hoped that these insights will allow research and clinical communities to engage more effectively with the severely affected CFS/ME population.

## 1. Introduction

Chronic Fatigue Syndrome/Myalgia Encephalomyelitis (CFS/ME) is a rare disease with no known etiology [1,2]. Its cause is unknown [3], and studies suggest it affects 0.4% of the population [4]. Criteria have been produced to identify clinical characteristics [5]; however, diagnosis remains by exclusion due to the absence of biomarkers [6], which has led to significant stigma [7].

Diagnostic criteria have evolved with better understanding of the condition. The International Consensus in 2011 [8] developed from a growing understanding of the condition and has led to the identification of heterogeneous groups within the CFS/ME population.

Subgroups had previously been defined by Cox et al. [9,10]. The categories are mild, moderate, severe, and very severe, they and were implemented in the National Institute for Health and Care Excellence guidelines. Severe and very severe CFS/ME individuals are wheelchair, house, or bedbound, due to the severity of their symptom burden.

The severe and very severe CFS/ME population find it difficult to access their wider environments. This creates difficulty for them to engage in research and healthcare provision as it is currently delivered. Therefore, this group are classed as hard to reach [11]. It is suspected that 10–25% of the 0.4% CFS/ME population are in the severe to very severe category. This figure has been supported by the CFS/ME charities and evidenced in the research literature [12,13]. It has been estimated that there are up to 40 people with CFS/ME in each GP practice across England, 25% of which are severe and very severe [14]. Therefore, across England alone, there are approximately 82,000 very vulnerable, severe, and very severely affected CFS/ME individuals housebound and bedbound that may not be currently not receiving appropriate treatment.

Quantitative and qualitative data are limited due to the hard to reach nature of the severely affected CFS/ME population. However, this is slowly changing, and texture is being added to the objective evidence published [15,16,17,18]. In recent years, more qualitative research has been performed with the CFS/ME population. However, the gap in knowledge regarding severely and very severely CFS/ME remains. Reviewing the research and evidence as it is published and reflecting on previous trials leads to a better understanding of how variables impact each other. The PACE trial (A randomized controlled trial of adaptive pacing, Cognitive Behavioural Therapy and graded exercise) was one such trial [19,20]. It used operational diagnostic criteria to identify 600 patients to be randomised to one of four treatment pathways: specialist care, plus or minus adaptive pacing therapy, Cognitive Behavioural Therapy (CBT), and Graded Exercise Therapy (GET). The aim was to gather scientific evidence to demonstrate the outcomes of each treatment. The research was planned and executed prior to the 2011 International Consensus Criteria [8], which identified the heterogeneity of the condition, categorising mild, moderate, severe, and very severe [9,10]. Within the PACE trial, the eligibility assessment and consent for treatment was the outcome 6-min walk test, which would have precluded severe and very severe patients from taking part in the study [21].

In addition to exclusion from major research, it is emerging that people with CFS/ME experience discrimination from healthcare professionals and wider society [14,22]. This can be partially attributed to a lack of understanding around the condition. CFS/ME is an illness, not a disease, and as such, it currently has no identifiable pathology [23]. This does not sit well within the current biomedical illness model that has been historically taught within medical schools [23]. Large institutions such as universities, hospitals, health, and social care agencies function within clear objective, evaluative models in which theories and ideas are critiqued and scrutinised. Evaluative models shift the physiological state to defence, which is incompatible with creativity and expansive theories [24].

People with CFS/ME that take a biomedical approach [23] may feel vulnerable without biomarkers to authenticate their illness or referral to specialist services to endorse complex overlapping symptoms [25]. The lack of identifiable pathology can result in psychological labelling or somatisation [23]. This lack of understanding can contribute to a sense of blame shifting, where patients may feel that they are held accountable for their poor health [23].

For therapy to work, an individual must first accept their situation [26] and feel believed [18]. This can be impeded when healthcare professionals, the structure they work within, and wider society do not acknowledge the limits of medical science and so continue to require hard evidence to support a diagnosis, rather than treat and manage symptoms [23,25,27,28]. The therapeutic relationship may be further jeopardised if an individual with CFS/ME does not have the energy required to express themselves effectively, particularly in a stressful situation where their illness may not be believed [18].

This article expands on a 2016 two-phase pilot study. The project aimed to understand the feasibility of severe and very severe CFS/ME individuals engaging with research, whilst scoping and defining the prevalence of CFS/ME in the region. In that study, 2.5% of the 2500 severe CFS/ME population in the northeast of England were identified and characterised. The study also explored the quality of life, symptom burden, and impact of severe CFS/ME, collating a database from a postal survey in phase 1 [29]. Using the database, five individuals with severe and very severe CFS/ME were identified for phase 2 of the study. Attending the participant in their own home to limit the research burden on the CFS/ME individual and aid pacing strategies is novel to this research area. The patient and public involvement and methodology for the entire study is presented elsewhere [29]. This paper presents findings from the five qualitative interviews.

### Aims

To explore the personal experience and understanding of individuals with CFS/ME.

To identify overarching themes that may highlight factors putting people at greater risk of experiencing the more severe presentation of CFS/ME.

To provide a better understanding of this population to allow healthcare and research communities to engage with individuals more effectively.

## 2. Materials and Methods

### 2.1. Methodology

A qualitative methodology involving in-depth interviews and drawing on phenomenology was selected. The interviews followed a semi-structured format [30], with interview schedules designed and worded to establish rapport and explore the area of concern in an open and flexible manner [31].

The aim of the interviews was to understand the perspective of the individual experience [32], to uncover personal meaning [31,33].

This group have limited presence in the research literature, and the methodology was explorative. By understanding the personal experience of individuals within the severe and very severe CFS/ME community, future qualitative research may be better focused with an improved understanding of practice, service, and research delivery to this population.

#### 2.1.1. Data Sampling

The sample was purposive [34], being defined as having severe CFS/ME as identified from the database of self-reported CFS/ME individuals. How the participants were recruited is described in some detail elsewhere [29]. However, for completeness, 483 questionnaire packs with an expression of interest (EOI) to be involved in future studies were posted out. There were 425 packs sent via the charity ME North East. Of those 483 packs, 63 were returned in various stages of completion. Within the returns were over 40 completed expression of interest forms to be involved in future studies.

Resource was a consideration when recruiting the five participants to understand the feasibility of engaging severely affected CFS/ME individuals with research. The returned EOI came from a large geographical area, which was to be covered by one researcher. The fluctuating and unpredictable health of the cohort was also considered. Therefore, participants were approached who lived near the research base to limit disruption if appointments had to be rescheduled at short notice. Participants within the designated area were contacted until five had been recruited. These participants each agreed to four home visits over a three-month period. In each case, the first visit was to obtain consent and perform autonomic tests; the second visit was cognitive testing; the third was an interview; and finally, there was a physical assessment.

The five participants completed a second consent form and patient information sheet, which was specific to phase 2 and detailed the agreement to have results published in a peer-reviewed journal.

The ethical principle of non-maleficence to do no harm [35] was a primary aim in planning the research.

#### 2.1.2. Data Collection

A pre-organised date and time was agreed, with the understanding that it could be rescheduled if necessary. Some participants gave specific instructions as to how to arrive at the home in order to reduce noise and stimulation.

Each interview lasted approximately one to two hours. Two patients had planned in advance and had compiled additional information to support the interview process and to reduce cognitive strain. A third patient found the study following a conversation too difficult and supplied a report that they had compiled over the course of their illness, which VS (author/researcher) read aloud as part of the interview, and the individual corrected or expanded on as they felt necessary. The youngest participant, who had been ill since her teens, requested that her mother be present to help answer any questions.

The interviews were conducted and recorded in conjunction with the collection of field notes [36,37,38]. This was to increase the trustworthiness and rigour of the data as well as the transferability of the findings [38].

#### 2.1.3. Data Analysis

Thematic analysis drew on a grounded theory approach, with a method of inductive analysis [39]. Data was coded without trying to fit it into a pre-existing framework. The researchers were implicit in the process, taking a constructivist style to develop theories based on the data, not a pre-defined question to answer. The aim was to explore the individuals’ experiences with severe CFS/ME.

Braun and Clark describe a six-phased approach that was followed [40]: initial familiarisation, generating codes, searching for themes, reviewing the themes and codes, defining and naming the codes, and finally writing the report. The software package NVivo version 12 was used to organise and analyse the data.

The transcripts were reviewed, and codes were created and grouped into similar themes. Then, themes were grouped for similarities and reduced to a manageable number.

Reflexivity was employed through conducting the analysis and interpretation of the findings with a second (qualitative) researcher (TC). The process was inductive, and the data produced were broad and rich.

### 2.2. Rigour

A research team with diverse background, knowledge, and skills was created to collect and analyse the data. The team had the collective ability to (1) access this hard-to-reach community, (2) collect the data whilst monitoring and limiting the impact the research might have on the participants, and (3) analyse the output. It was through this pooling of skills that this research was made possible. The potential for research bias is recognised and was limited through co-author collaboration of the research team to increase the trustworthiness of the analysis [38].

A clinically reasoned decision was taken not to have participants validate the researcher’s transcripts. It is understood that “member checking” increases the internal validity and credibility of research [38]. However, this was outweighed by the need to adhere to the ethical principle of non-maleficence, to do no harm [35]. Participants were very fragile, and so it was felt that additional home visits would impact health and function too greatly. However, the Chief Executive Officer of the charity ME North East, who was instrumental in accessing the interviewed individuals, was sent a summary of the findings on behalf of the participants.

### 2.3. Ethical Considerations

Full ethics approval was granted by North East-Newcastle and North Tyneside 2 Research Ethics Committee. The participants had provided separate informed consent for both phase one and two of the study.

Pseudonyms have been given to each of the participants to protect their identities and maintain confidentiality

## 3. Results

### 3.1. Findings

The characteristics of the five participants are presented in Table 1. Individual circumstances created very diverse presentations, despite each participant being within the severe or very severe CFS/ME category.

Three overarching themes were identified from the initial codes. Within two of these themes, subthemes were identified (see Table 2).

Each of these themes is now discussed in turn.

#### 3.1.1. Theme 1: Lived Experience

The illness experiences of each of the five participants were very different, with one shared feature: the impact the illness had had on their lives. This overarching ‘Lived Experience’ theme explores the experience of living with severe CFS/ME and incorporates two subthemes: history and initial presentation and the impact the illness has had on each participant.

#### 3.1.2. History and Initial Presentation

This subtheme considers family history, comorbidities, life events, age at onset of illness, initial presentation, and advice. The participants became ill at different points in their life, with different resources and burdens to manage their illness with. This led to different expressions of the illness, reflecting diverse lives and values and unique personal biopsychosocial frameworks.

For all the individuals, there was a recognised pre-existing vulnerability to becoming ill. Then, a trigger led to the development of multiple symptoms. This is illustrated in Table 3.

There was also evidence that for some participants, precipitating behaviours and circumstances made managing this complex cluster of symptoms that define the illness difficult. For several, it was an active life. For example, Lorraine became ill after completing her honours degree. She continued to struggle, living alone whilst trying to establish a new career and complete a postgraduate degree. Similarly, Jane was a busy full-time working mum of a sick baby who required regular hospital admissions.

For the individuals who became ill as young adults, multiple burdens were not a factor; however, they had not created robust coping strategies to manage a debilitating long-term condition before the illness was triggered. As can be seen from Table 1, David and Helen did not have the opportunities to establish themselves in a workplace or higher education to gain life experience before circumstances called on them to manage their illness.

Two of the five participants expressed how the initial presentation of this illness was difficult to describe to healthcare professionals, as there were often multiple competing and overlapping symptoms. For example, Abi described feeling *“tired exhausted, muscles were hurting. Felt poisoned, more than ill, horrible feeling all over, like that, but I was just feeling weird and wrong.”*

When the illness was triggered in a transitionary phase of life e.g., new jobs/careers, new parents, it was difficult to understand the cause and effect. Jane stated: *“In hindsight, it wasn’t a normal type of tiredness. I just didn’t have the words or assertiveness to convince anyone.”*

The initial presentation was often vague, and the initial advice received from healthcare professionals was often to keep going, stay active and get fitter.

Most participants described a “persisting” or “boom or bust” behaviour pattern during a sustained period in which they attempted, unsuccessfully to regain their previous “normal”. For example, Jane changed jobs, trying to get a healthier balance: *“I took a month off, when I was to get myself well, get myself fit. Doctors’ advice was to exercise in the hope that, you know, start of the new job physically better, physically fitter.”*

Helen was given similar advice, and being younger, it was her mum who directed the activity: “I did try taking her to the park, running around, desperately thinking she needs to exercise, she needs exercise. And then she was wiped out for days and days and days.” Exercise intolerance appears to be present for multiple individuals; however, without an objective physical cause, it is difficult to identify. Each of the participants deteriorated whilst trying to get fitter or be “normal”.

There appears to be a consistent pattern of boom/bust and persistence leading to deterioration, with consequent periods of bedrest to alleviate symptoms, resulting in deconditioning and limited function, which can be profound.

At best, energy fluctuations allow individuals to experience only fleeting moments of “normal”. For example, Jane stated she was active for 2% of the day when she washed and toileted, whereas Lorraine was completely bedbound and dependent on assistance just to sit up in bed.

For all or many of the participants, tasks such as personal hygiene and food preparation are limited to basic needs. Overlaying complexities of orthostatic symptoms, allergies, and sensitivities all impact activities of daily living. This was demonstrated by David being limited to a bowl of cornflakes on the days his ageing mother could not assist him to make a meal, which was becoming more frequent. Jane reluctantly admitted she could possibly make a meal for herself twice a month and at times had gone days without food when her carers had been away; she was simply too ill to do any more than the essentials. Nutrition had been prioritised out of that energy calculation.

Sleep was also affected. Two individuals described parasomnias, sleeping 22 h per day for long periods of time or being awake but unable to move. Lorraine related her poor sleep to autonomic disturbance: *“hyperadrenergic-over heating and waking hourly. Bad dreams, hallucinations”.*

Jane expressed that sleep was beyond her control due to family circumstances, with *“two small children who can be ill and climb into bed”.*

### 3.2. Impact on Life

This subtheme illustrates the impact on life including function, nourishment, sleep, and social isolation. Individuals acknowledge the confining effects of their illness. Some found it difficult to live with meaning, as this required energy and effort that they did not have. Therefore, life was a passage of time, without having the resources to take action. For David, *“The biggest problem for people with ME, you are in limbo, nobody knows what to do with you. What life, because it’s more about filling time than actually living, because living requires doing things with much effort.”*

However, Jane had adapted and altered her thoughts to accept her situation, prioritising her energy for the activities that gave her life meaning and value, demonstrating resilience to her situation:

*“I have all the people and things that are important in life and you know, like, relationships and the love you have got in your family, I still have that so my life has been boiled down to the most important bits that are still here. If I was going to lose things from my life, it would be my work, and yeah, it would be reluctantly to be able to go outside and have a life, but it’s these special relationships that I cherish that make me feel happy and content.”*

#### 3.2.1. Theme 2: Challenges to Everyday life

This overarching theme comprises two subthemes: Intrinsic—those elements that were fundamental to the individual’s physiology or psychology and extrinsic—those elements that operated from outside the individual.

#### 3.2.2. Intrinsic Concepts

Within this subtheme were three distinct concepts. The physical concept included the effect of activity, adrenaline response/orthostatic intolerance, allergies, and sensitivities and baseline level of activity/unpredictable fluctuations. Processing/psychological concept encompassed belief about cure, personal views, views about management, communication, and energy balance. Finally, there were concepts comprising cognitive impairment, dissociation/detachment, and mental health.

Each individual’s ability to function was limited to their personal capacity, which fluctuated extremely and created difficulties to plan and manage basic day-to-day activities such as washing, toileting, and in some instances feeding, particularly with the added symptom of allergies and sensitivities. For example, Lorraine said, *“Digesting food is an energy challenge,”* and Abi found, *“In the food department (allergies), the big one is nickel, which limits food diversity.”*

It appears that when baseline energy levels are so low and capacity is so limited, there is a frequent tendency to experience the challenge of everyday activities as a physiological threat. Participants often access the sympathetic nervous system just to carry out basic functions such as personal hygiene and eating. It seems this often leads to dysautonomia, which is a disruption of the autonomic nervous system that regulates the heart, blood vessels, digestion, and breathing. This disruption could be due to activity and/or orthostatic challenge. For example, Jane said, *“I am in bed 100% of the time. When active, pain feels worse, feel weak, feeling hot, cold, fevery, kind of heart palpitation, breathlessness, shaky.”*

The management of physiological and physical limitations was often bound by thoughts and perceptions of the participant’s experience, which was in turn often limited by their condition. The ability to process and communicate beliefs about their situation were limited by their energy levels. Lorraine said:

*“You get to a point in a relationship where you actually need to say, ‘This is what is going on with my illness...’ And then you have to eat and we never get a chance, there’s no time for conversation. All my emotions are around, are set to one side, all the loss, all the bereavement, loss of self, loss of life, loss of opportunity, loss of the living, so family relationships are just set to one side, there isn’t time to process those emotions to ever have them.”*

The processing ability appeared to be further impacted by cognitive impairments. Individuals recognised this; however, the strategies they put in place created increased energy expenditure.

Compounding these challenges was the concerning presence of dissociation and detachment, which was expressed by four of the five participants, along with, in some cases, loss of identity. For example: *“I feel detached and confused. I suffer from disconnectedness, so I don’t feel physically present—don’t concentrate on that—having a blister and gritting your teeth. I don’t know, I can’t remember”* (Lorraine). *“There is numbness most of the time, a contentedness. Then the rest of the time—frustration, disappointment, fear. My body being unresponsive. Just staring into space. I don’t feel myself anymore, sense of self or identity... I have accepted, peaceful, but I struggle with I don’t feel like myself anymore” (Jane).*

#### 3.2.3. Extrinsic Concepts

This subtheme relates to the influences outside of the individual’s control that harm their ability to function in the world given their illness. Such influences include the benefits system, lack of professional understanding, and prejudicial views.

Most social challenges came from a lack of understanding within society as to the nature of CFS/ME or the extent to which it can impact. This lack of understanding can lead to prejudicial views and preconceptions that impede an individual’s access to healthcare and social services. For example, Abi conveyed an experience she had had in an accident and emergency department, having been taken from her health centre to hospital in an ambulance. Once she arrived, the attitude of the staff changed when they realised she was a frequent visitor: *“I passed out. I woke up and was being dragged along the floor by two orderlies and this nurse screaming at me.”*

Abi received the care she required once her tachycardia was identified. Lorraine, too, recognised this engrained culture. *“It takes years to erode institutionalised discrimination and prejudice which are unhealthy and negative for both victim and perpetrator.”*

Health and social care require a diagnosis to be current in order for an individual to be eligible for social support. This support is binary: there is no grading within the system. This creates a difficulty when there is not a biomedical marker to identify the illness. The repeated cycle of having to demonstrate ill health impacts an individual’s ability to manage and improve from that health issue. It also undermines continuity of recognition of that ongoing issue. For example, Lorraine said, *“I lack a current diagnosis so I can’t get my benefits. They keep saying no current diagnosis.”* Similarly, for David, *“After probably a year to a year and a half of having ME, I was improving, doing much better, but then the benefits agency reviewed and decided I was fit for work. I lasted 6 months working as admin in a restaurant before I crumbled… [following repeated appeals the benefits withdrawal decision was repealed]. I should never have been taken off (benefits) in the first place.”*

#### 3.2.4. Theme 3: Management of the Condition

This theme incorporates managing and coping strategies, relief from symptoms, understanding acceptance, acceptance of professional lack of understanding, social media, GP attitudes, and healthy carer beliefs.

The five individuals interviewed managed their condition within the confines of the means at their disposal, both intrinsic and extrinsic. Their strategies were founded on a subjective understanding of their capabilities, their illness, and their resources. Participants relied on support from family members, social media, and the internet to gain information they required. Social media was used to maintain contact with friends. For example, Abi used *“Twitter with friends”* and Helen used the Internet as she transitions from sleep to wakefulness, *“In bed 30 min am on phone waking up, googling”.*

Whilst the internet alleviates the social isolation, it can distil and reinforce beliefs. For example, Abi reported, “I have never been to a CFS clinic and I am glad, because I won’t want to do GET (Graded Exercise Therapy). I know from experience, physically pushing past what you feel, it made me worse, so I wouldn’t want to entertain that.”

In terms of social support, Helen described how she was reliant on the availability of her working parents to take her out to socialise. However, their availability also had to coincide with when she was well enough. Helen’s parents appeared to have had sacrificed their social life to prevent her from being left alone and isolated. Her family appeared to plan their lives around her illness, as far as they could whilst also maintaining their income. The focussed pressure on caregivers was substantiated by Jane, who explained it was her husband, the income provider, who worked full time, was the main caregiver to two primary aged children, and was also her care giver. Therefore, it was not only the CFS/ME participants who had reduced their lives to necessary priorities but also their caregivers and families.

In addition to social support, healthcare professionals were acknowledged as people who could help.

Abi reported the occasional doctor who understands, whilst Helen and her mother were very keen to praise their current GP as “fabulous” because they understood Helen’s situation. This relieved a lot of stress. Lorraine found that individuals who were not fixed in their beliefs of the condition were most beneficial: *“People who are genuine, non-judgemental and open minded”* (Lorraine).

This often left a very narrow path to navigate. In turn, this may limit the potential for an individual with CFS/ME to improve. For example, Abi stated “I have learnt it is pointless, some people won’t listen and there is no point. I have tried to fight back and been called neurotic. I was really frustrated, not neurotic.” Similarly, for David reported:

*“Resignation—it’s not a great surprise after all these years. You hear about how much progress they make with this and that and the other and you think yeah, but there is obviously an awful lot of conditions where nothing changes for decades.”*

## 4. Discussion

Our first aim was to explore the personal experience and understanding of individuals with CFS/ME. This was achieved through open questioning and exploration of the participant’s views of their reality.

The second aim was to identify overarching themes that may help identify risk factors that place people at greater threat of experiencing the more severe presentation of CFS/ME. Many of the participants demonstrated previously identified risk factors for expressing the severe form of CFS/ME: a delayed diagnosis [22,28]; problems accessing social security [22] and poor relationships with doctors or health professionals [14,22,23].

This small qualitative study has identified other common factors, which need further research to clarify and confirm. For example, demonstration of deterioration as the individual initially attempted to get fitter or remain “normal”. This may be an indication of an unidentified exercise intolerance. It appears that the point in a person’s life when the illness presents is of importance. Several participants were moving from one phase of life to another. For example, school exam time, moving from school or higher education to work life, or following the birth of a child. It appears that severe presentation may manifest when the illness coincides with a transitioning time in a person’s life. Another common factor is the relationship between burden and resource. Those with dependents or many responsibilities and a limited support network appear to be more vulnerable to the severe expression of the illness. In addition, those individuals who had a support network but remained to some degree dependent on carers were not able to establish independence due to the illness.

The final aim of this study was to provide a better understanding of this population to allow a research community to engage with them more effectively. This has been addressed to an extent by Kingdon [14]. We have taken a phenomenological approach to report the lived experience of five individuals with CFS/ME. These findings cannot be generalised; however, it is possible that they are transferable to other individuals in a similar position. Here, we will expand on how these findings may be applied to the evolving understanding.

All five of the participants had vague initial presentations that they found difficult to explain, illustrating the experience of living with poorly understood illness. Despite a fatigue presentation, they were actively encouraged to keep going and push through or had themselves tried to regain their former life. Maladaptive sickness patterns have been recognised in chronic illness [41], and the recommendation of exercise in the presence of fatigue is increasingly acknowledged as detrimental. Inappropriate advice may promote unhealthy pacing behaviours of “boom and bust” and persistence [42]. It is suggested that this ultimately leads to deconditioning through the over training exercise curve, which is recognised in athletes but remains an under researched area [43]. Fatigue self-efficacy improves outcomes [41]; however, the confidence to self-manage fatigue must be fostered gradually and immediately if unhealthy adaptive behaviours are to be avoided.

The timing of the illness appears to have importance. When illness occurs at a young age, school attendance is reduced, seriously affecting intellectual and social development [16,44]. This is illustrated by Helen and to some extent David, who continued to be dependent on their parents, who were their carers into adulthood. This combination can further impact managing this complex illness [15]. Another critical factor is if the illness occurs during a transition, e.g., from professional to working mother, when tiredness is expected. This may make diagnosis more difficult: transitioning life stages produce confounding factors that confuse a biomedical assessment.

All the participants followed a deteriorating pattern. It appears there comes a point when burdens exceed resources and the opportunity to improve is extinguished. Then, people experience physiological threats, resulting in fight or flight reactions or dissociative responses. Dissociation is described within polyvagal theory as losing a sense of presence resulting in experiencing a disconnection and a lack of continuity between thoughts, memories, surroundings, and actions [45].

It is concerning that at least two of the participants reported symptoms of dissociation. It is suspected in the three others. Benign aspects of existence were experienced as threats so extreme that the ability to be present was lost. Acceptance has been identified as a precursor for any therapeutic intervention to succeed. However, it is proposed that for acceptance to occur, a person must feel safe and present within their physical environment. This has significant implications for management and rehabilitation.

Intrinsic challenges to everyday life are further compounded by the extrinsic burdens. All five of the participants reported poor or limited interactions with healthcare professionals during their illness. Negative attitudes towards CFS/ME by medical professionals are repeatedly reported [23,46]. After many attempts at trying and failing to navigate the health and social care systems, with an imbalance of energy, resources, and burdens, some individuals experiencing CFS/ME eventually appear to accept their limitations and those of their health professionals.

All of the participants were forced to give up education or employment. In work and educational institutions, the lack of understanding and provision for people with CFS/ME creates obstacles for people with the illness to remain in those environments. CBT and GET do not restore the ability of a person with CFS/ME to work [47]. People with CFS/ME who cannot remain in employment need to access the benefits system. As CFS/ME does not sit within the current biomedical model of health and social care, this creates issues navigating the benefits system. This was reported by two of the five participants. It has been recorded that the benefits system in the UK does not meet the needs of people with CFS/ME, leaving them socially isolated and/or increasingly dependent on friends and family. The distress of navigating the system often exacerbates health conditions [40].

The five participants managed their condition as well as resources allowed, both intrinsic and extrinsic. At times, this unfortunately meant accepting the limits of the system in which they found themselves.

Four of the five study participants had received specialist support during their illness. However, this support was not always valued. One participant was receiving support at the time of the study. All the participants presented with complex multi-faceted issues that impacted every component of the biopsychosocial model. Their ability had declined to the extent where it impacted every aspect of their life: physical function, diet, sleep, and social interaction.

The participants were heavily reliant on the internet to source management strategies. This often distilled illness beliefs. Health literacy has been shown to be a challenge in vulnerable groups [48]. However, we do not understand how severely affected CFS/ME individuals use health literature, because they are so under researched.

It appears that severely affected CFS/ME individuals must lead a very disciplined and limited existence in order to manage symptom burden within their intrinsic and extrinsic limitations. It is an open question as to whether such limits impact their ability to be psychologically flexible and resilient in their outlook.

## 5. Conclusions

This study is novel, as it has accessed this hard to reach population group and recorded their experience. Most of the participants had received some form of specialist CFS/ME support or had access to the healthcare services. However, their experiences ranged from accepting the limitations of the service to having a very negative view.

CFS/ME is a medically unexplained illness lying at the boundaries of understanding within the legacy biomedical model. An illness where there is no single, simple cause or theoretical model, no clear mind/body division, and no definitive classification [1] does not sit easily in the current healthcare system. The CFS/ME presentation conflicts with the current health and social care model [1,2]. The severe CFS/ME presentation sits outside the model and therefore is not acknowledged.

This illness ranks low within primary care, as it is not life threatening [23]. However, it is potentially life shortening [14]. There are certainly physical and mental health symptoms that are often disregarded or missed within the complex presentation [14], and reports suggest that 88 suicides have been partly attributed to CFS/ME between 2001 and 2016. However, it has been noted that it is not necessarily intrinsic factors that lead to suicide, but a combination of extrinsic factors, which include a lack of medical care and social support, failure to control key symptoms, and inadequate financial help. Depression is not always a feature in CFS/ME-related suicide [49].

Pathway-focused institutional cultures are not predisposed to embrace the ambiguities inherent in adopting the more holistic biopsychosocial model, where outcomes are more difficult to define and evaluate. The resulting continued narrow biomedical focus of the current social care system results in neither the healthcare professional nor the CFS/ME patient feeling safe with each coming from a position of defence when they communicate [23,27,45]. People with the severe expression of CFS/ME appear to avoid the harm of the current health and social care system by purposely withdrawing from it. This reduces opportunities for rehabilitation and is an area for further study.

Individuals with severe CFS/ME live on the peripheries of society, at the edges of the research bell curve [50]. They do not belong within “normal” expectations and they do not have the energy to try to fit [51]; therefore, they remain socially, medically, and financially isolated. The role of environment has been discussed within the international classification of function. Disability has been acknowledged as a socially created problem that can limit freedom by failure to provide the resources and opportunities needed to make participation feasible [52]. This paradigm must be explored further if we are to better understand and provide adequate health and social care for the severe CFS/ME population or other people experiencing “illness” that does not fall into the biomedical model.

The findings of this study aim to assist understanding of the needs of the severe CFS/ME population. Currently, the healthcare system and research community are failing to provide resources and opportunities for this group to engage, and so enable the positive outcome of increased independence. Longer periods of intervention, home visits and telephone consultations and in extreme cases inpatient rehabilitation in specialist services are effective evidenced interventions in the research literature [10,16,44,53]. Such services would meet the needs of CFS/ME individuals much better than the status quo which often forces patients to meet the needs of the system in order to secure the care that they need.

A re-evaluation of the approach taken to CFS/ME and other unexplained illness is ever more urgent given the upcoming surge in numbers of long-haul COVID-19 individuals. A major symptom of such long-haul COVID-19 is fatigue [54,55]. Research and healthcare communities have much experience to share and further research to perform, particularly in the area of health, social care, and societal attitudes allowing vulnerable ill people to remain valued members of society.

### Limitations

The thematic analysis aspect of this research studies a small number of participants in depth, giving a rich presentation. The participants were from a small geographical area and may not be representative of the wider CFS/ME community.

It is recommended that further research is conducted with a larger sample of participants across a wider geographical area of the United Kingdom. Adequate financial and time provision must be allocated to allow severe and very severe CFS/ME individuals to engage in future projects. Part of future research regarding CFS/ME must explore the wider biopsychosocial factors that lead to the severe expressions of fatigue. The goal is to identify risk factors that affect the deterioration of the condition within different life phases and aid earlier detection of those at risk of the severe and very severe expression of CFS/ME and adequate provision of healthcare.

## Figures and Tables

**Table 1:** Participant characteristics.

Pseudonym	Age Range	Length of Illness	Gender	Living Arrangements	Support	Dependents	Background
JANE:	36–44	7 years	Female	Terraced house, predominantly bedbound, upstairs toilet.	Husband and mother	2 primary aged children	Very physically active prior to illness, professional, mother of 2 married.
DAVID:	36–45	18 years	Male	Semi-detached house. Predominantly housebound, living mostly in his bedroom.	Ageing parents	0	Single. Completed A-levels and managed to do office work between episodes of ill health, until early 20s. Had been very active prior to ill health.
ABI:	36–45	20 years	Female	Lived in a bungalow. Predominantly house bound.	Husband and ageing parents nearby	0	Married. Had been very active prior to ill health.
LORRAINE:	56–65	37 years	Female	Bungalow. Completely bedbound in a bedroom.	24-h social care	0	Single, professional, unable to work. Had been very active academically prior to ill health.
HELEN:	16–25	6 years	Female	Lived in a house with parents and older sister. Housebound, except for occasional outing assisted by family.	Working age parents and an older sister	0	Single, education abandoned due to ill health. Not able to work or continue education due to symptoms burden.

**Table 2:** Themes and subthemes.

Theme	Subtheme	Concepts within Subtheme
Lived experience	History and initial presentation	
	Impact of illness	
Challenges to Everyday Life	Intrinsic	Physical
		Processing/Psychological
		Cognitive
	Extrinsic	
Management		

**Table 3:** Characteristics of early illness.

Pseudonym	Pre-Existing Vulnerability	Trigger	Initial Symptoms	Time to Diagnose	Transitions of Coping Strategy
Jane	Young mum, full-time worker, transitioning point in life.	Complex. Viral infections, birth of son.	Myalgia, pain, viral infections.	3 years	Persisting to Acceptance
David	Never returned to pre-glandular fever energy levels. Began working full time. Contracted influenza B. Transitioning point in his life.	Influenza B.	Post viral fatigue, aches and pains.	3 months to diagnose post viral fatigue.	Avoidance ‘Head in the sand like an ostrich’.
Abi	Not listening to body.	Tick bite. Working as a gym and aerobics instructor. Just kept going.	Overly hot and sweating during routine exercise. Exhausted, muscles hurting, fatigue.	Unknown	Persisting to acceptance of situation.
Lorraine	Limited support network. Establishing herself in her new career. Transitioning point in her life	Viral.	Orthostatic intolerance, fatigue, weakness.	Unknown	Persisting and boom and busting towards acceptance.
Helen	Exam time, transitioning point in her life.	Migraines and hypersomnia	Headaches, migraines, and hypersomnia	Unknown	Degree of persisting, boom and busting due to limited experience.

## Data Availability

The data presented in this study are available on request from the corresponding author. The data are not publicly available due to privacy and data protection protocols.
